# Supplementary material for: Recruitment of the mitotic exit network to yeast centrosomes couples septin displacement to actomyosin constriction
Source: Nat Commun. 2018 Oct 17;9:4308. doi: 10.1038/s41467-018-06767-0 (PMC6193047; doi:10.1038/s41467-018-06767-0)
Supplement: Supplementary file 2 — Description of Additional Supplementary Files [file 41467_2018_6767_MOESM2_ESM.pdf]

Description of Additional Supplementary Files

File Name: Supplementary Movie 1
Description: Constitutive recruitment of Cdc14 to SPBs suppresses the cytokinetic defects of GAL1-DMA2 cells. GAL1-DMA2 BUD4 cells expressing Nud1-GBD at endogenous levels and Cdc14-GFP from a centromeric plasmid were imaged at 30°C every 4 min in selective medium (-His containing raffinose and galactose) after being induced for ~90 min with galactose. Left panel: transmitted light; central panel: Shs1-mCherry; right panel: Cdc14-GFP.

File Name: Supplementary Data 1
Description: List of *S. cerevisiae* strains used in this study (plasmids are indicated in brackets)
