# Supplementary material for: Recruitment of the mitotic exit network to yeast centrosomes couples septin displacement to actomyosin constriction
Source: Nat Commun. 2018 Oct 17;9:4308. doi: 10.1038/s41467-018-06767-0 (PMC6193047; doi:10.1038/s41467-018-06767-0)
Supplement: Supplementary file 4 — Supplementary_dataset [file 41467_2018_6767_MOESM4_ESM.docx]

**Table S1. List of *S. cerevisiae* strains used in this study** (plasmids are indicated in brackets)

**Name** **Relevant genotype**

ySP52 *MATalpha, cdc15-2*

ySP285 *MATalpha, cdc14-3*

ySP311 *MATalpha, dbf2-2*

ySP325 *MATalpha, cdc5-2::URA3*

ySP1515 *MATa, nud1::S.p.HIS5, trp1-1::TRP1::nud1-44*

ySP3016 *MATa, ura3::URA3::GAL-DMA2s^a^*

ySP3018 *MATa, ura3::URA3::GAL-DMA2s^b^*

ySP3309 *MATa, DMA2-3HA::K.l.URA3*

ySP4014 *MATa, cdc5-2::URA3, ura3::URA3::GAL-DMA2s^a^*

ySP7217 *MATa, leu2::LEU2:TEM1-Q79L*

ySP7349 *MATa, ura3::URA3::GAL-DMA2s^b^, leu2::LEU2:TEM1-Q79L*

ySP9863 *MATa, TEM1-3HA::KlURA3, [CUP1-Ubi in YEp96 (TRP1)]*

ySP9864 *MATa, TEM1-3HA::KlURA3, [CUP1-6His-Ubi in YEp96 (TRP1)]*

ySP9865 *MATa, TEM1-3HA::KlURA3, dma2::hphMX, dma1::K.l.LEU2, [CUP1-Ubi in YEp96 (TRP1)]*

ySP9866 *MATa, TEM1-3HA::KlURA3, dma2::hphMX, dma1::K.l.LEU2, [CUP1-6His-Ubi in YEp96 (TRP1)]*

ySP10394 *MATa, TEM1-eGFP::kanMX, SPC42-mCherry::natMX*

ySP10472 *MATalpha, tem1-3*

ySP10550 *MATa, CDC15-GFP::kanMX, SPC42-mCherry::natMX*

ySP11334 *MATa, bud4::LEU2::BUD4*

ySP12398 *MATalpha, SHS1-mCherry::hphMX, MYO1-GFP::kanMX, bud4::LEU2::BUD4*

ySP12406 *MATa, SHS1-mCherry::hphMX, MYO1-GFP::kanMX, bud4::LEU2::BUD4, ura3::URA3::GAL1-DMA2m^b^*

ySP12418 *MATa, trp1::TRP1::CDC14^TAB6-1^, bud4::LEU2::BUD4, SHS1-mCherry::hphMX, MYO1-GFP::kanMX, dbf2-2*

ySP12498 *MATa, bud4::LEU2::BUD4, SHS1-mCherry::hphMX, MYO1-GFP::kanMX, dbf2-2*

ySP12508 *MATa, SHS1-mCherry::hphMX, MYO1-GFP::kanMX, bud4::LEU2::BUD4*

ySP12643 *MATa, shs1::natMX, CDC10-6Gly-eGFP::kanMX, bud4::LEU2::BUD4*

ySP12716 *MATa, SHS1-mCherry::hphMX, MYO1-GFP::kanMX, bud4::LEU2::BUD4, trp1::TRP1::CDC14^TAB6-1^, ura3::URA3::GAL1-DMA2m^b^*

ySP12805 *MATalpha, SHS1-mCherry::hphMX, MYO1-GFP::kanMX, bud4::LEU2::BUD4, cdc14-3*

ySP12809 *MATa, SHS1-mCherry::hphMX, MYO1-GFP::kanMX, bud4::LEU2::BUD4, tem1::GAL1-UPL-TEM1::TRP1*

ySP12875 *MATa, SHS1-mCherry::hphMX, MYO1-GFP::kanMX, bud4::LEU2::BUD4, ura3::URA3::GAL1-DMA2m^b^, [TEM1-Q79L in YCplac22 (TRP1)]*

ySP12975 *MATa, SPC42-mCherry::natMX, CDC5-eGFP::KanMX*

ySP13042 *MATa, DMA2::GAL-DMA2::URA3m^b^, bud4::LEU2::BUD4, shs1::natMX*

ySP13115 *MATalpha, DMA2::GAL-DMA2::URA3m^b^, cdc12-1, SHS1-mCherry::hphMX, MYO1-GFP::kanMX, bud4::LEU2::BUD4*

ySP13134 *MATa, SHS1-mCherry::hphMX, MYO1-GFP::kanMX, bud4::LEU2::BUD4, tem1::GAL1-UPL-TEM1::TRP1, trp1::TRP1::CDC14^TAB6-1^*

ySP13140 *MATa,* *bud4::LEU2::BUD4, DMA2::GAL-DMA2::URA3m^b^*

ySP13163 *MATa, bud4::LEU2::BUD4, shs1::natMX*

ySP13167 *MATa, bud4::LEU2::BUD4, shs1::natMX, MYO1-GFP::kanMX, ura3::URA3::GAL1-DMA2m^b^*

ySP13174 *MATa, TEM1-eGFP::kanMX, SPC42-mCherry::natMX, ura3::URA3::GAL1-DMA2m^b^*

ySP13229 *MATa, SHS1-mCherry::hphMX, MYO1-GFP::kanMX, bud4::LEU2::BUD4, trp1::TRP1::CDC14^TAB6-1^, cdc15::CDC15-as1(L99G)::URA3*

ySP13259 *MATa, SHS1-mCherry::hphMX, MYO1-GFP::kanMX, bud4::LEU2::BUD4, cdc15::CDC15-as1(L99G)::URA3*

ySP13261 *MATa, TEM1-3xHA::K.l.URA3, ura3::URA3::GAL1-DMA2m^b^, [CUP1-Ubi in YEp96 (TRP1)]*

ySP13262 *MATa, TEM1-3xHA::K.l.URA3, ura3::URA3::GAL1-DMA2m^b^, [CUP1-6His-Ubi in YEp96 (TRP1)]*

ySP13351 *MATa, CDC15-GFP::kanMX, SPC42-mCherry::natMX, ura3::URA3::GAL1-DMA2m^b^*

ySP13416 *MATa, NUD1-3PK::K.l.HIS3*

ySP13447 *MATa, NUD1-3PK::K.l.HIS3, [CUP1-Ubi in YEp96 (TRP1)]*

ySP13448 *MATa, NUD1-3PK::K.l.HIS3, [CUP1-6XHis-Ubi in YEp96 (TRP1)]*

ySP13449 *MATalpha, dma2::hphMX, dma1::K.l.LEU2, NUD1-3PK::K.l.HIS3, [CUP1-Ubi in YEp96 (TRP1)]*

ySP13450 *MATalpha, dma2::hphMX, dma1::K.l.LEU2, NUD1-3PK::K.l.HIS3, [CUP1-6His-Ubi in YEp96 (TRP1)]*

ySP13528 *MATa, NUD1-3PK::K.l.HIS3, ura3::URA3::GAL1-DMA2m^b^*

ySP13549 *MATa, dma2::K.l.LEU2, dma1::K.l.TRP1, CDC15-GFP::kanMX, SPC42-mCherry::natMX*

ySP13554 *MATa, NUD1-3PK::K.l.HIS3, ura3::URA3::GAL1-DMA2m^b^, [CUP1-Ubi in YEp96 (TRP1)]*

ySP13555 *MATa, NUD1-3PK::K.l.HIS3, ura3::URA3::GAL1-DMA2m^b^, [CUP1-6His-Ubi in YEp96 (TRP1)]*

ySP13575 *MATa, NUD1-GBD::kanMX*

ySP13629 *MATa, cdc15-2, ura3::URA3::GAL-DMA2s^a^*

ySP13631 *MATa, dbf2-2, ura3::URA3::GAL-DMA2s^a^*

ySP13634 *MATalpha, cdc14-3, ura3::URA3::GAL-DMA2s^a^*

ySP13653 *MATa, tem1-3, ura3::URA3::GAL-DMA2s^a^*

ySP13670 *MATa, nud1::S.p.HIS5, trp1::TRP1::nud1-44, SHS1-mCherry::hphMX, MYO1-GFP::kanMX, bud4::URA3::BUD4*

ySP13683 *MATa, nud1::S.p.HIS5, trp1-1::TRP1::nud1-44, ura3::URA3::GAL-DMA2s^a^*

ySP13719 *MATa, SPC42-mCherry::natMX, CDC5-eGFP::kanMX, ura3::URA3::GAL1-DMA2m^b^*

ySP13729 *MATa, bud4::HIS3*

ySP13731 *MATa, mob1::HIS3::GFP-MOB1::URA3, SPC42-mCherry::natMX*

ySP13733 *MATa, dma2::K.l.LEU2, dma1::K.l.TRP1, mob1::HIS3::GFP-MOB1::URA3, SPC42-mCherry::natMX*

ySP13735 *MATa, SPC42-mCherry::natMX, BFA1-eGFP::kanMx*

ySP13747 *MATa, ura3::URA3::GAL1-DMA2m^b^, leu2::LEU2:TEM1-Q79L, bud4::LEU2::BUD4*

ySP13749 *MATa, leu2::LEU2:TEM1-Q79L, bud4::LEU2::BUD4*

ySP13751 *MATa, mob1::HIS3::GFP-MOB1::URA3, SPC42-mCherry::natMX, ura3::URA3::GAL1-DMA2m^b^*

ySP13758 *MATa, SPC42-mCherry::natMX, BFA1-eGFP::kanMx*

ySP13772 *MATa, SHS1-mCherry::hphMX, MYO1-GFP::kanMX, bud4::HIS3, ura3::URA3::GAL1-DMA2m^b^*

ySP13777 *MATa, trp1::TRP1::CDC14^TAB6-1^, bud4::LEU2::BUD4, SHS1-mCherry::hphMX, MYO1-GFP::kanMX, dbf2-2, dbf20::natMX*

ySP13859 *MATa, ura3::URA3::GAL1-DMA2m^b^, cdc12-1, bud4::LEU2::BUD4*

ySP13860 *MATa, cdc12-1, bud4::LEU2::BUD4*

ySP13887 *MATa, bud4::LEU2::BUD4, ura3::URA3::GAL1-DMA2m^b^, shs1::natMX, CDC10-6Gly-eGFP::kanMX*

ySP14042 *MATa, trp1::TRP1::CDC14^TAB6-1^, ura3::URA3::GFP-CDC12, bud4::LEU2::BUD4, dbf20::natMX, dbf2-2-1XminiAID::kanMX, [GAL1-OsTIR1 in pRS313 (CEN, HIS3)]*

ySP14043 *MATa, trp1::TRP1::CDC14^TAB6-1^, ura3::URA3::GFP-CDC12, bud4::LEU2::BUD4, dbf20::natMX, dbf2-2-1XminiAID::kanMX, [GAL1-OsTIR1 in pRS313 (CEN, HIS3)]*

ySP14044 *MATa, trp1::TRP1::CDC14^TAB6-1^, ura3::URA3::GFP-CDC12, bud4::LEU2::BUD4, dbf20::natMX, dbf2-2-3XminiAID::kanMX, [GAL1-OsTIR1 in pRS313 (CEN, HIS3)]*

ySP14045 *MATa, trp1::TRP1::CDC14^TAB6-1^, ura3::URA3::GFP-CDC12, bud4::LEU2::BUD4, dbf20::natMX, dbf2-2-3XminiAID::kanMX, [GAL1-OsTIR1 in pRS313 (CEN, HIS3)]*

ySP14057 *MATalpha, SHS1-mCherry::hphMX, MYO1-GFP::kanMX, bud4::LEU2::BUD4, trp1::TRP1::CDC14^TAB6-1^, mob1-77*

ySP14087 *MATa, ura3::URA3::GAL1-DMA2m^b^, SHS1-mCherry::hphMX, bud4::LEU2::BUD4, [CDC14-GFP-HisMX6 in pRS315 (CEN, LEU2)]*

ySP14119 *MATalpha, bud4::LEU2::BUD4, CDC14-6Gly-eGFP::kanMX*

ySP14120 *MATa, ura3::URA3::GAL1-DMA2m^b^, bud4::LEU2::BUD4, CDC14-6Gly-eGFP::kanMX*

ySP14129 *MATa, NUD1-GBD::kanMX, ura3::URA3::GAL1-DMA2m^b^, bud4::LEU2::BUD4, SHS1-mCherry::hphMX, [CDC14-GFP-HisMX6 in pRS315 (CEN, LEU2)]*

ySP14130 *MATa, NUD1-GBD::kanMX, ura3::URA3::GAL1-DMA2m^b^, bud4::LEU2::BUD4, SHS1-mCherry::hphMX, [pRS313 (HIS3)]*

ySP14131 *MATa, NUD1-GBD::kanMX, bud4::LEU2::BUD4, SHS1-mCherry::hphMX, [CDC14-GFP-HisMX6 in pRS315 (CEN, LEU2)]*

ySP14132 *MATa, NUD1-GBD::kanMX, bud4::LEU2::BUD4, SHS1-mCherry::hphMX, [pRS313 (HIS3)]*

ySP14316 MATa, *SHS1-mCherry::hphMX, MYO1-GFP::kanMX, bud4::LEU2::BUD4, mob1-77*

ySP14339 *MATa, CDC11-HA::HIS3, ura3::URA3::GAL1-DMA2m^b^, [CUP1-Ubi in YEp96 (TRP1)]*

ySP14340 *MATa, CDC11-HA::HIS3, ura3::URA3::GAL1-DMA2m^b^, [CUP1-6His-Ubi in YEp96 (TRP1)]*

ySP14341 *MATa, SHS1-HA::HIS3, ura3::URA3::GAL1-DMA2m^b^, [CUP1-Ubi in YEp96 (TRP1)]*

ySP14342 *MATa, SHS1-HA::HIS3, ura3::URA3::GAL1-DMA2m^b^, [CUP1-6His-Ubi in YEp96 (TRP1)]*

ySP14355 *MATa, ura3::URA3::GAL1-DMA2m^b^, SHS1-mCherry::hphMX, CHS2-GFP::HIS3, bud4::LEU2::BUD4*

ySP14357 *MATa, SHS1-mCherry::hphMX, CHS2-GFP::HIS3, bud4::LEU2::BUD4*

ySP14362 *MATalpha, ura3::URA3::GAL1-DMA2m^b^, SHS1-mCherry::hphMX, IQG1-GFP::LEU2, bud4::LEU2::BUD4*

ySP14364 *MATa, SHS1-mCherry::hphMX, IQG1-GFP::LEU2, bud4::LEU2::BUD4*

ySP14490 *MATa, NatN2::GALs-DMA2-eGFP::KanMX*

ySP14496 *MATa, NUD1-GBD::kanMX, NatN2::GALs-DMA2-eGFP::KanMX*

ySP14497 *MATalpha, NUD1-GBD::kanMX, NatN2::GALs-DMA2-eGFP::KanMX, SHS1-mCherry::hphMX*

ySP14498 *MATa, NUD1-GBD::kanMX, NatN2::GALs-DMA2-eGFP::KanMX, SHS1-mCherry::hphMX, bud4::LEU2::BUD4*

ySP14533 *MATa,* *SHS1-mCherry::hphMX, MYO1-GFP::kanMX, bud4::LEU2::BUD4, , trp1::TRP1::CDC14^TAB6-1^, mob1-77*

ySP14666 *MATa, DMA2-3HA::K.l.URA3, NUD1-6Gly-3Flag::KanMX*

^a^: all strains marked *GAL1-DMA2s* carry one single copy of the *GAL1-DMA2* construct integrated in the genome

^b^: all strains marked *GAL1-DMA2m* carry multiple copies (≥3) of the *GAL1-DMA2* construct integrated in the genome. All these strains are derived by genetic crosses and therefore carry the same copy number of integrated *GAL1-DMA2.*
