# Supplementary material for: Recruitment of the mitotic exit network to yeast centrosomes couples septin displacement to actomyosin constriction
Source: Nat Commun. 2018 Oct 17;9:4308. doi: 10.1038/s41467-018-06767-0 (PMC6193047; doi:10.1038/s41467-018-06767-0)
Supplement: Supplementary file 5 — Supplementary_info_v2 [file 41467_2018_6767_MOESM5_ESM.pdf]

Supplementary Information

Recruitment of the Mitotic Exit Network to yeast centrosomes couples septin displacement to actomyosin constriction

Tamborrini et al.

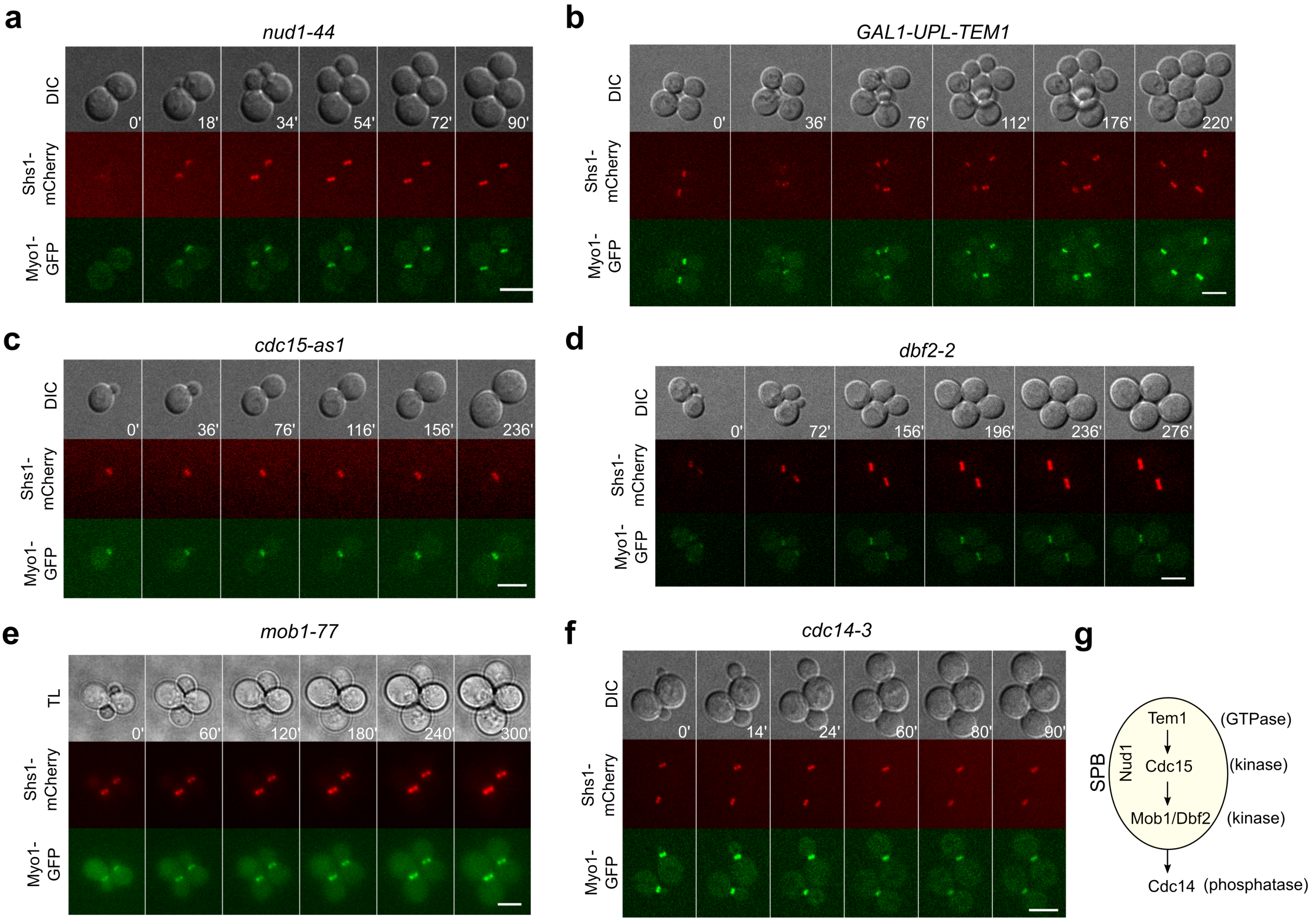

Supplementary Figure 1. MEN is necessary for septin ring splitting and CAR constriction
a-f: Cells with the indicated genotypes (all *BUD4*) and expressing Shs1-mCherry and Myo1-GFP were grown in permissive conditions and then shifted to restrictive conditions 60-90 min prior to imaging. Cells were filmed every 2-4 min for 4 hours in restrictive conditions (a: 34°C; b: glucose-containing medium; c: medium supplemented with 5μM 1NM-PP1; d: 34.5°C; e: 32°C; f: 37°C). DIC: differential interference contrast; TL: transmitted light. Scale bar: 5 μm. **g:** The cartoon represents the hierarchical recruitment of MEN factors to SPBs, with the Nud1 protein acting as scaffold. Note that once released from the nucleolus Cdc14 also localises to bud-directed SPB, partly recruited by the GAP Bub2-Bfa1 that in turn requires Nud1 for its SPB localisation (not depicted).

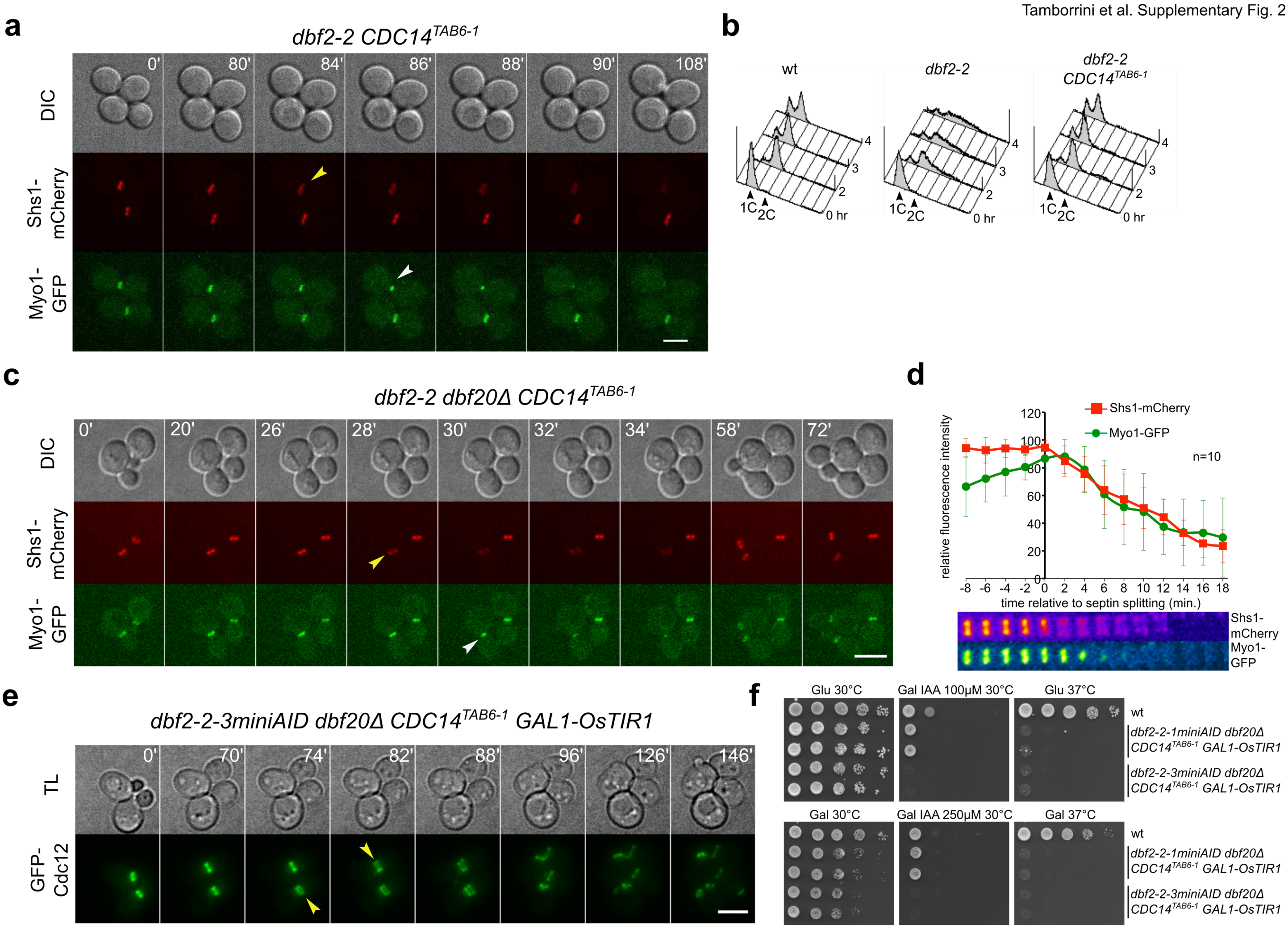

Supplementary Figure 2. The redundant Dbf2 and Dbf20 kinase may be dispensable for septin ring splitting

a, c, e: Cells with the indicated genotypes (all *BUD4*) and expressing Shs1-mCherry along with Myo1-GFP were grown in permissive conditions and then shifted to restrictive conditions 60-90 min prior to imaging. Cells were filmed every 2 min (a, c) or 4 min (e) in restrictive conditions (a: 34.5°C; c: 35.5°C; e: galactose-containing medium supplemented with 250 μM indoleacetic acid (IAA) at 30°C; similar data to e) were obtained by treating cells with 100 μM naphtaleneacetic acid (NAA) at 30°C). DIC: differential interference contrast. TL: transmitted light. Scale bar: 5 μm. **b:** Cells with the indicated genotypes were grown in YEPD at 25°C, arrested in G1 by alpha factor and then released in fresh YEPD at 34.5°C for FACS analysis of DNA contents. FACS data were plotted after gating out the debris as illustrated in Supplementary Fig. 12. **d:** Quantification of fluorescence intensities associated to Shs1-mCherry (red squares) and Myo1-GFP (green circles) in the cells filmed in (b) around the time of septin ring splitting (time 0). Average data from 10 different cells are plotted ± s.d. (error bars). We note that under these conditions CAR constriction occurred almost concomitant to septin ring splitting, suggesting that the temporal separation between the two events is attenuated. Cropped images beneath the graph show the behaviour of septin ring and CAR during this time frame in one representative cell. **f:** Serial dilutions of cells with the indicated genotypes were spotted on glucose- or galactose containing plates in the absence or presence of IAA. Plates were then incubated at the indicated temperatures.

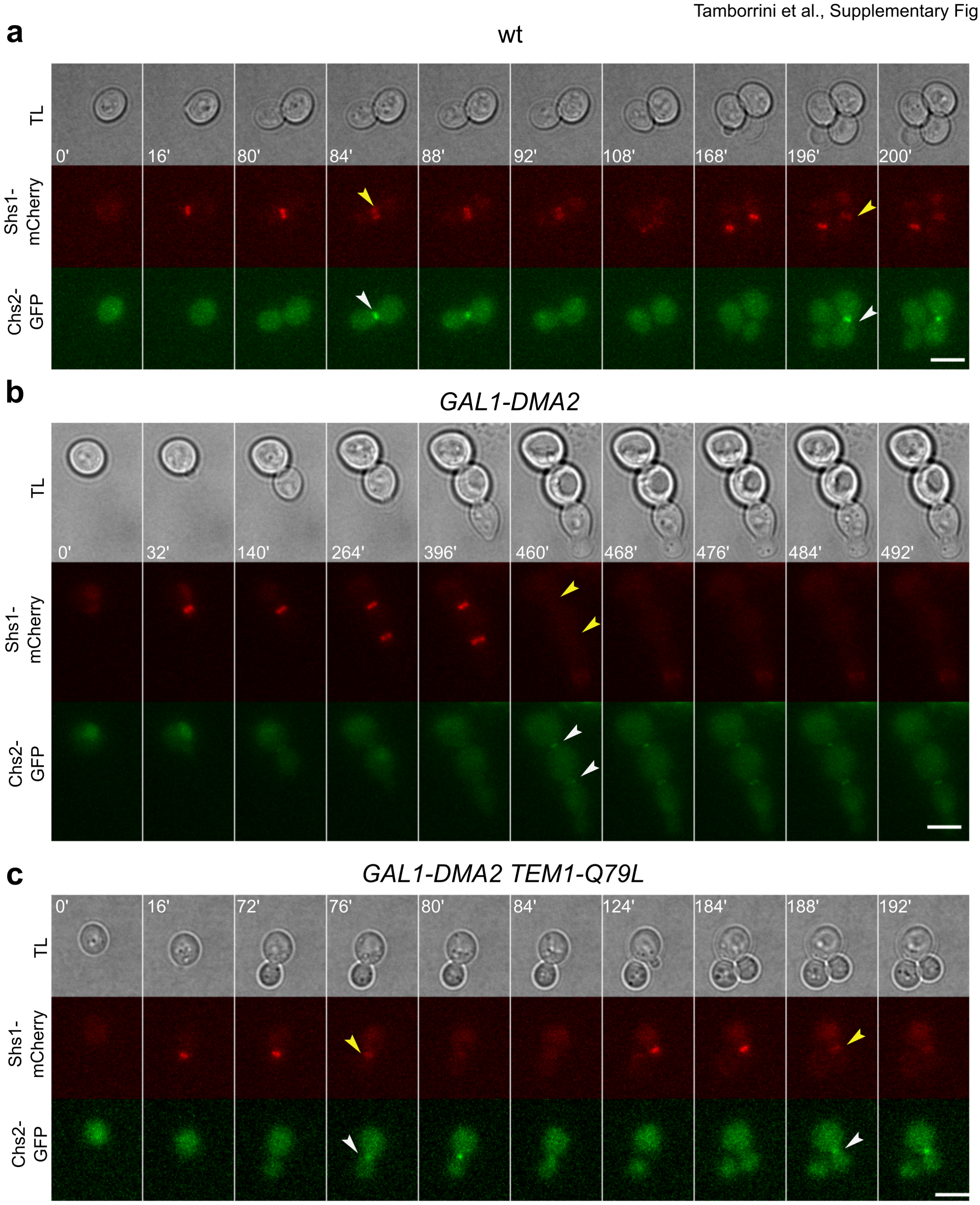

d

	septin displacement Chs2 at bud neck	septin displacement no Chs2 at bud neck	stable septins Chs2 at bud neck	stable septins no Chs2 at bud neck
wild type	139	0	0	0
GAL1-DMA2	58	5	0	64
GAL1-DMA2 TEM1-Q79L	141	11	0	14

Supplementary Figure 3. Bud neck recruitment of the chitin synthase Chs2 does not occur upon inhibition of septin ring splitting
a-d: Cells with the indicated genotypes and expressing Shs1-mCherry and Chs2-GFP were grown in SD-raffinose and induced for 90 min with galactose before being mounted with SD-raffinose/galactose for imaging at 30°C every 4 min. Scale bar: 5 μm. The number of cells categorized according to Chs2 recruitment to the bud neck in relation to septin behaviour is shown in (d). Yellow arrowheads: septin splitting/clearance; white arrowheads: Chs2 recruitment to the neck. We note that late disappearance of septins after mitotic exit in *GAL1-DMA2* cells is sometimes accompanied by recruitment of low levels of Chs2 to the bud neck (b).

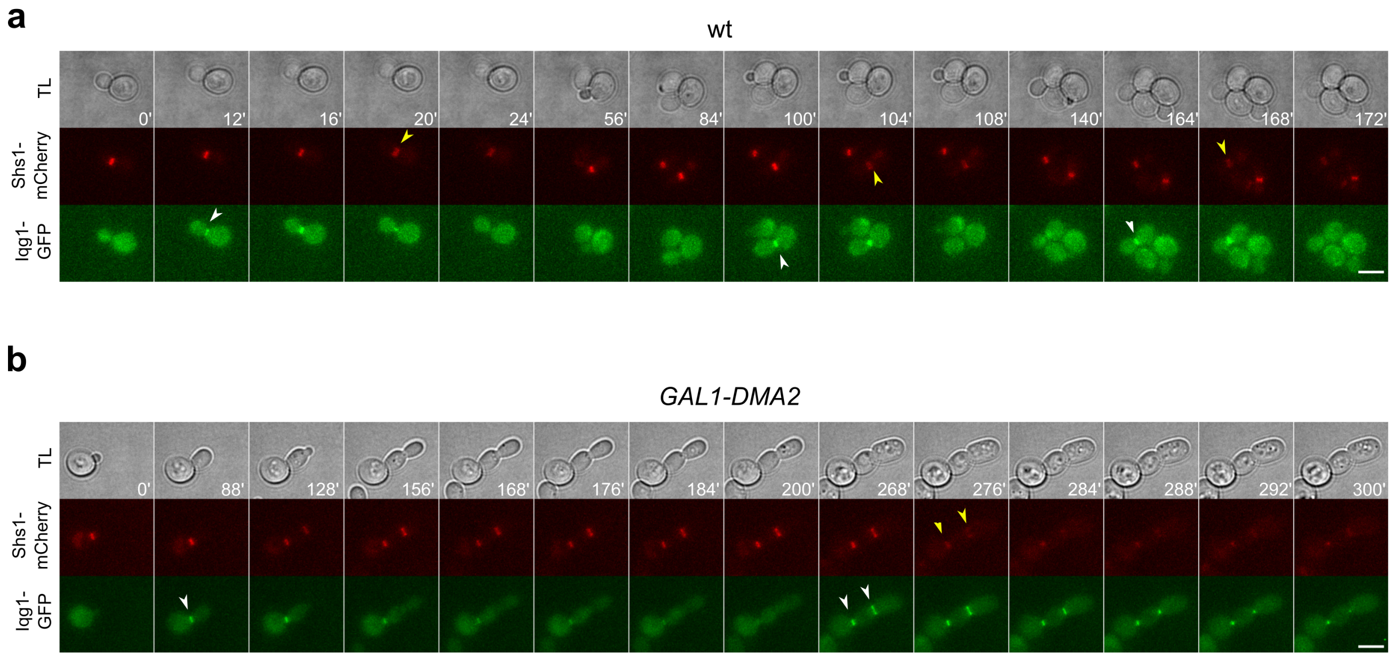

Supplementary Figure 4. The IQGAP Iqg1 is normally recruited to the bud neck upon *DMA2* overexpression

Wild-type and *GAL1-DMA2* cells expressing Shs1-mCherry and Iqg1-GFP were grown in SD-raffinose and induced for 90 min with galactose before being mounted with SD-raffinose/galactose for imaging at 30°C every 4 min. Iqg1-GFP was recruited to the bud neck in late mitosis in 100% of the cells (n=155 for wild-type (a) and n=156 for *GAL1-DMA2* (b)). We note that in *GAL1-DMA2* cells the Iqg1 ring, although not constricting, gradually faded away (156'-200') presumably through APC^{Cdh1}-dependent degradation [1], followed by its reappearance at both necks marked by unsplit septin rings (268'). Around 276' septins were cleared from the bud neck and the Iqg1 rings slowly constricted (276'-300'). Scale bar: 5 μm.

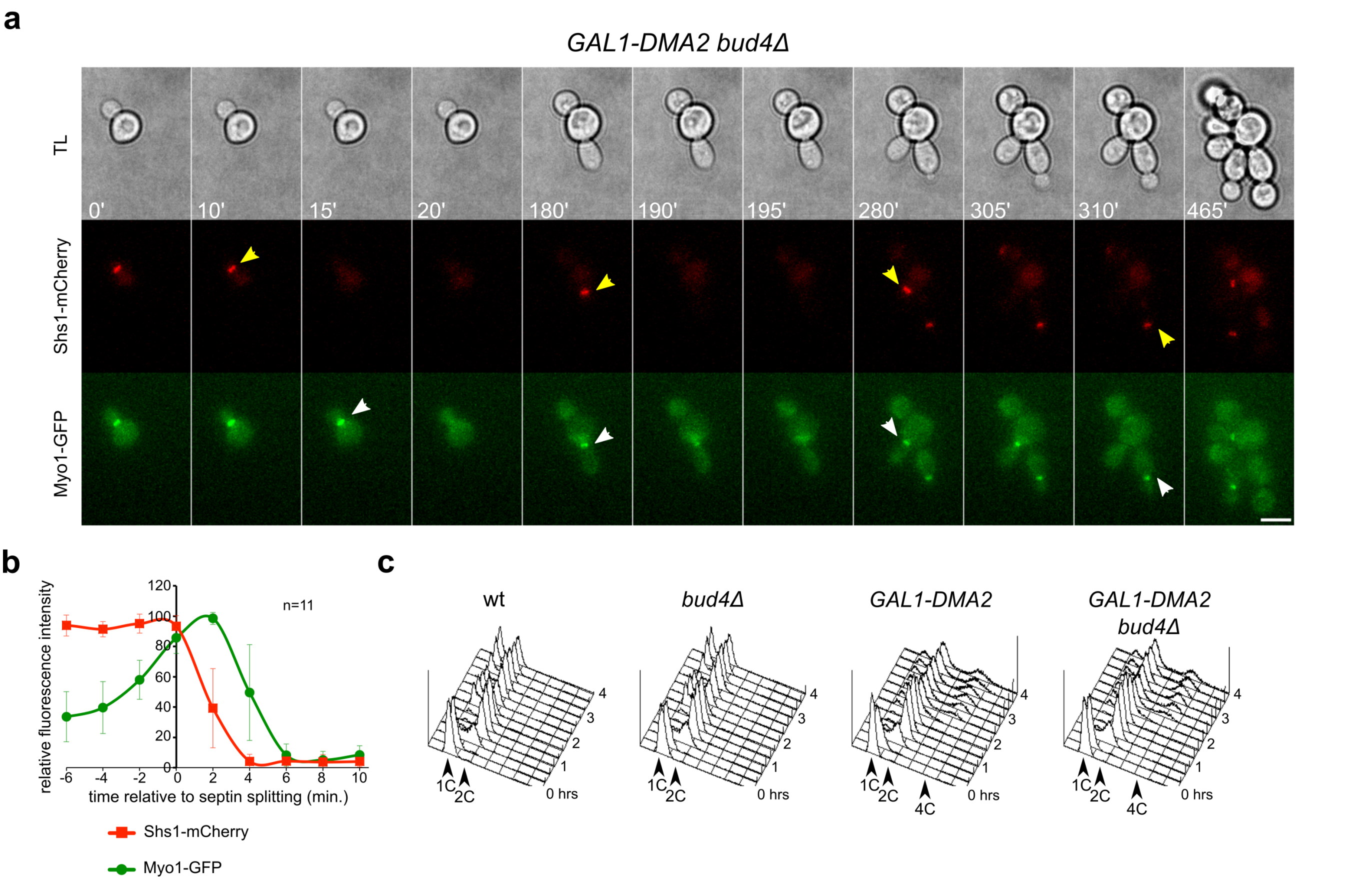

Supplementary Figure 5. *BUD4* deletion allows septin disappearance and CAR constriction in *GAL1-DMA2* cells but does not fully rescue their cytokinesis defects

a: *GAL1-DMA2 bud4*Δ cells expressing Shs1-mCherry and Myo1-GFP were grown in SD-raffinose and induced for 90 min with galactose before being mounted with the same medium for imaging at 30°C every 5 min. Arrowheads indicate septin rings before disappearance (yellow) or CARs before constriction (white). Scale bar: 5 μm. **b:** Quantification of fluorescence intensities associated to Shs1-mCherry (red squares) and Myo1-GFP (green circles) in cells filmed in (a) around the time of septin ring disappearance (time 0). Average values from 11 different cells are plotted ± s.d. (error bars). **c:** Cells with the indicated genotypes were grown in YEPR at 25°C, arrested in G1 by alpha factor and induced by galactose 30 min before the release. After release in YEPRG at 25°C (time 0) cell samples were withdrawn at the indicated time points for FACS analysis of DNA contents. FACS data were plotted after gating out the debris as illustrated in Supplementary Fig. 12.

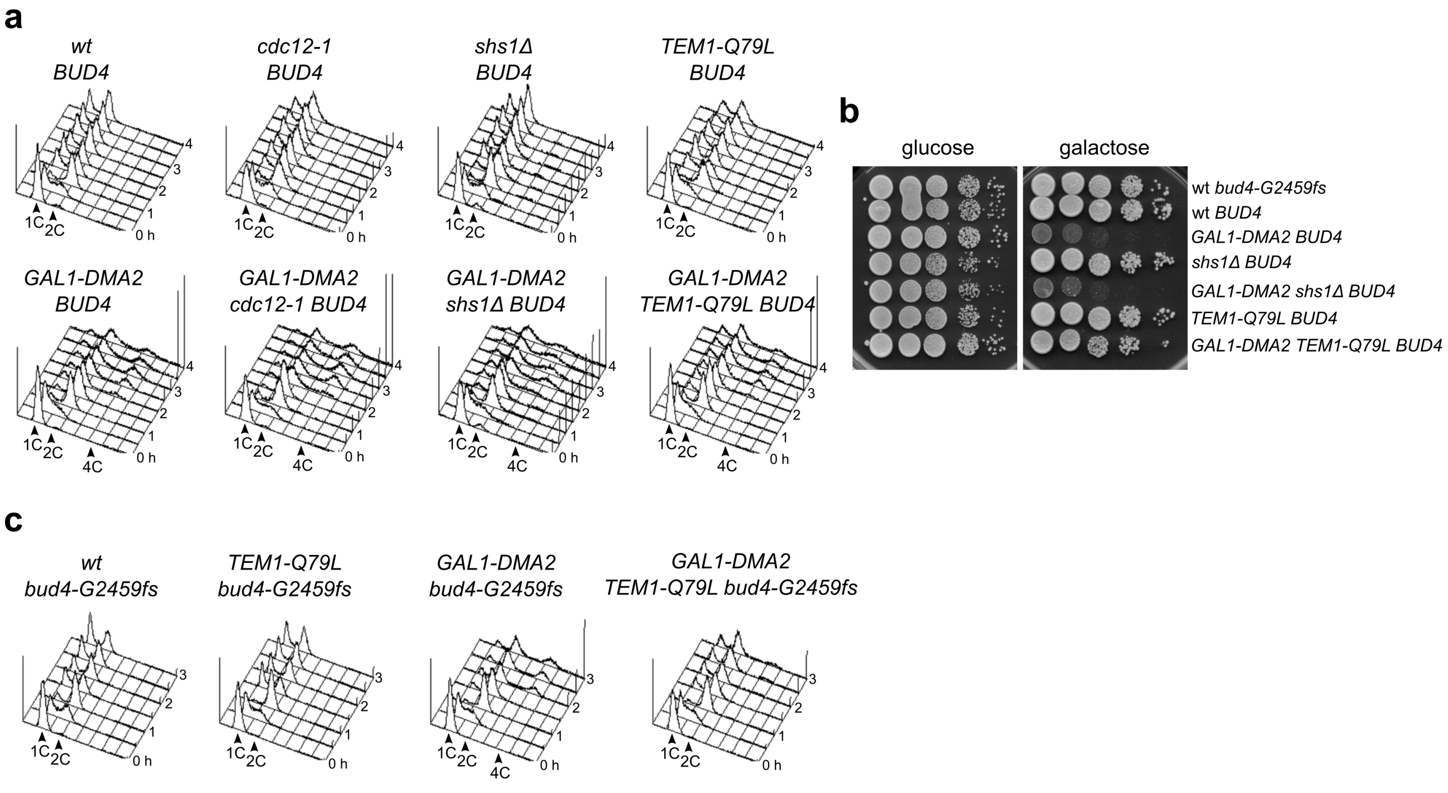

Supplementary Figure 6. MEN hyperactivation restores complete cytokinesis and cell separation upon *DMA2* overexpression in a *bud4-G2459fs* but not in *BUD4* background

a, c: Cells with the indicated genotypes were grown in YEPR at 23°C, arrested in G1 with alpha factor, induced with galactose 30' prior to release and finally released in YEPRG medium at 30°C. At different time points after release (time 0) cell samples were withdrawn for FACS analysis of DNA contents. FACS data were plotted after gating out the debris as illustrated in Supplementary Fig. 12. **b:** Serial dilutions of cells with the indicated genotypes were spotted on YEPD (glucose) and YEPG (galactose) plates and incubated at 30°C.

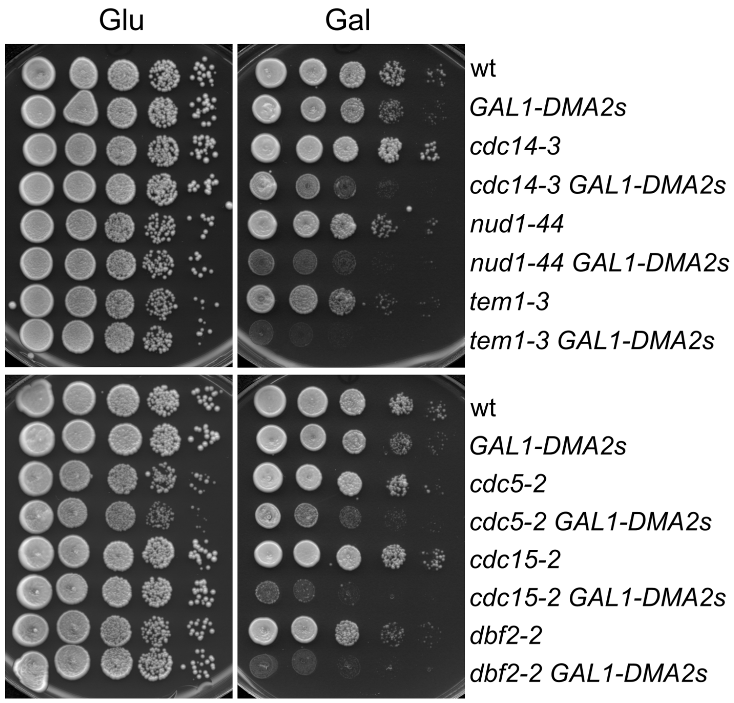

Supplementary Figure 7. Moderate *DMA2* overexpression is toxic for MEN mutants at permissive temperature

Serial dilutions of cells with the indicated genotypes were spotted on YEPD (Glu) and YEPG (Gal) plates and incubated at 25°C. We refer to moderate overexpression of *DMA2* as that obtained with one single copy of the *GAL1-DMA2* construct integrated at the *URA3* locus (*GAL1-DMA2s*). All experiments reported elsewhere in this manuscript relied on heavier overexpression obtained by integration of multiple copies of integrated *GAL1-DMA2* (≥3, [2]).

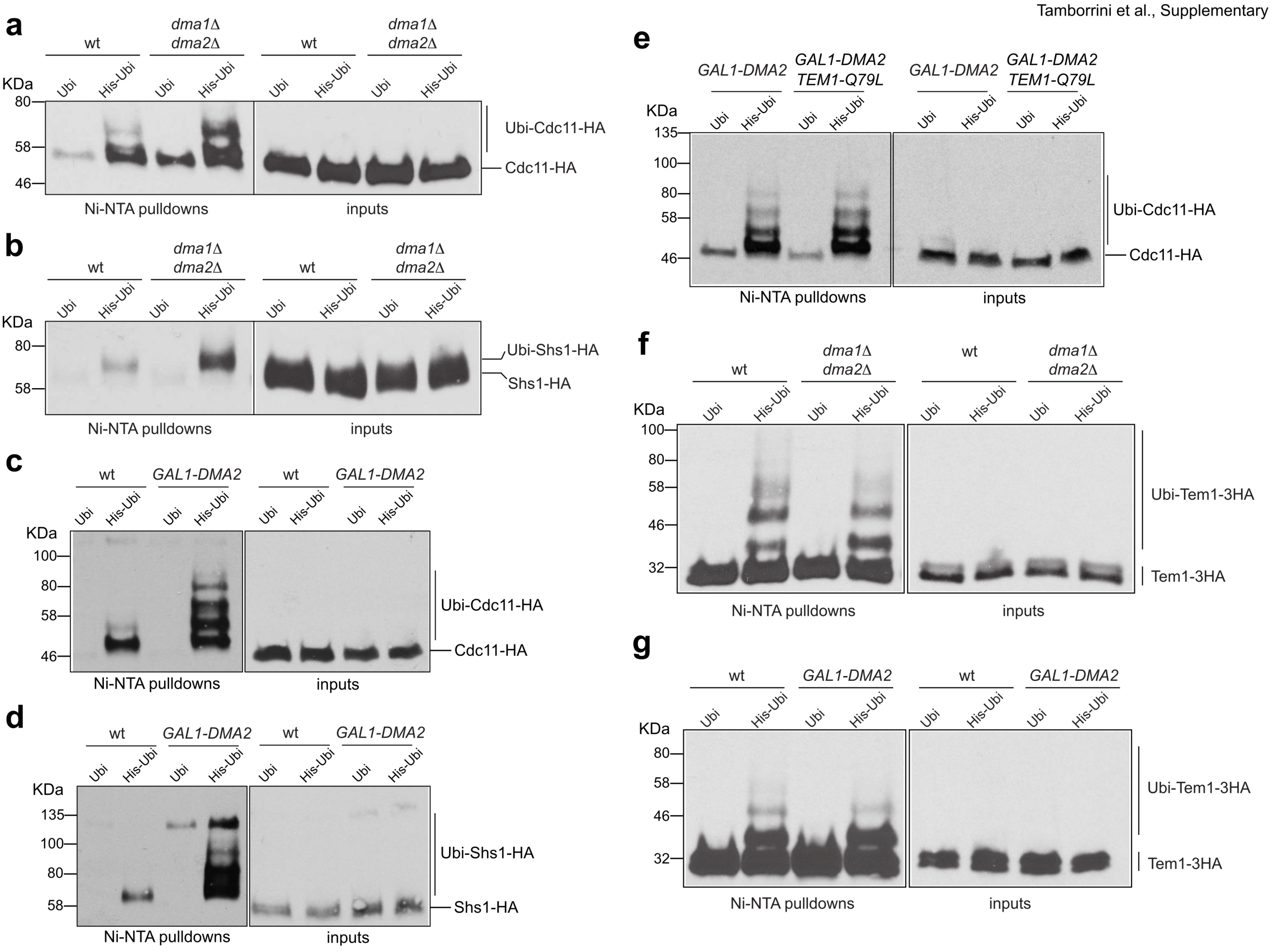

Supplementary Figure 8. Dma1 and Dma2 are not necessary for septin or Tem1 ubiquitination
a-g: Ni-NTA pulldown assays were carried out using cell extracts from strains with the indicated genotypes expressing Cdc11-HA, Shs1-HA or Tem1-3HA at endogenous levels and overexpressing either untagged ubiquitin or His-tagged ubiquitin from the *CUP1* promoter. Ubiquitination of the three proteins was revealed by western blot using an anti-HA antibody. *DMA2* was overexpressed for 30' by addition of 1% galactose to raffinose-containing medium (c, d, e, g).

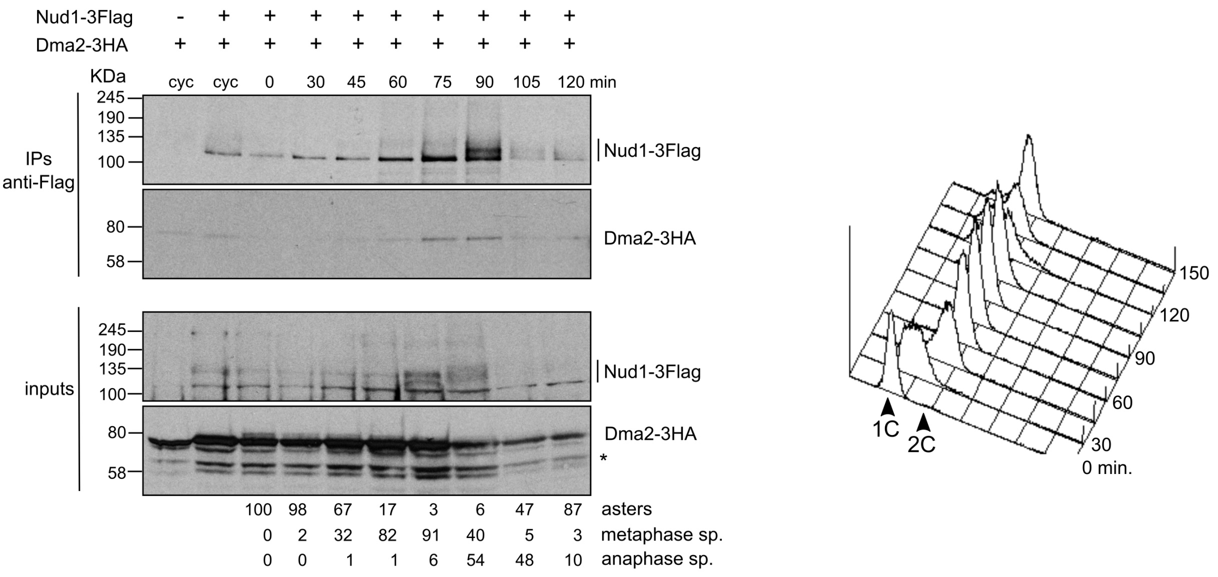

Supplementary Figure 9. A small fraction of Dma2 interact physically with Nud1 in mitosis
Cells co-expressing Nud1-3Flag and Dma2-3HA were grown in YEPD at 25°C, arrested in G1 by alpha factor and then released in fresh YEPD at 25°C (time 0). Cell samples were withdrawn at different time points to assess Nud1-Dma2 interaction by co-immunoprecipitations, as well as for FACS analysis of DNA contents (right histograms) and tubulin immunofluorescence (sp.: spindles). Inputs represent 1/40th of the lysates used for immunoprecipitations. The asterisk indicates a degradation product. FACS data were plotted after gating out the debris as illustrated in Supplementary Fig. 12.

a

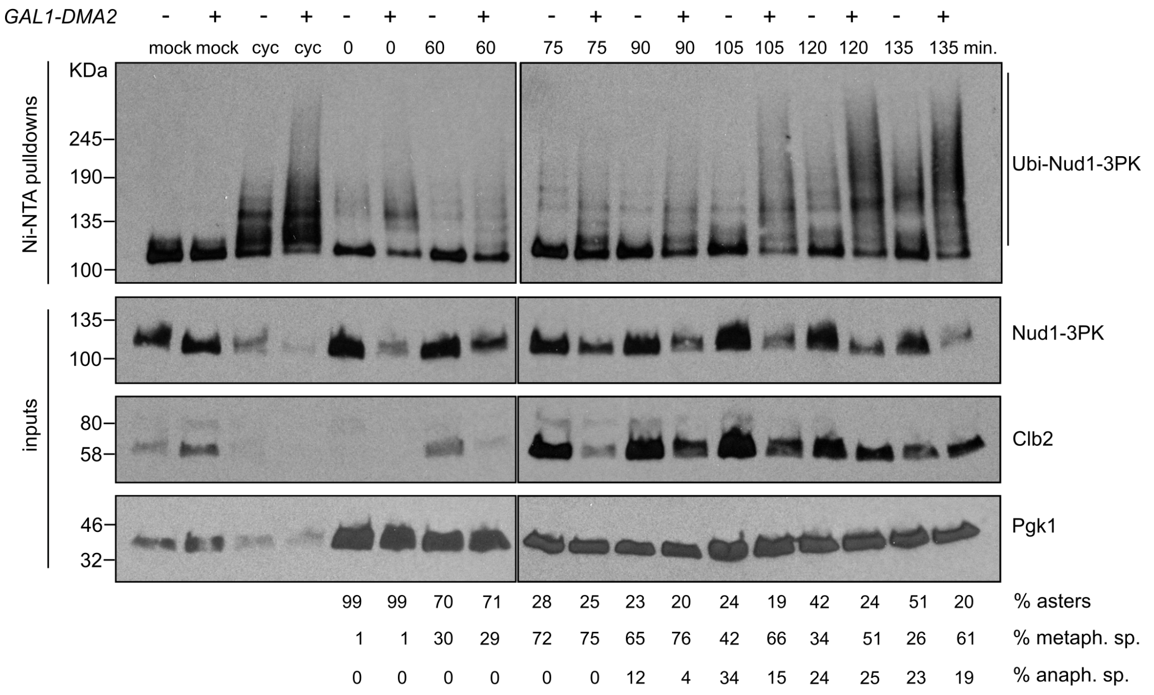

b

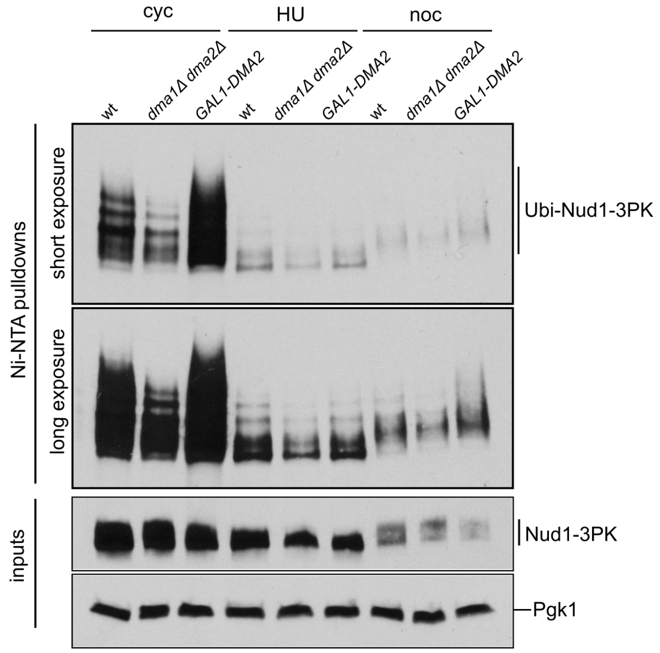

Supplementary Figure 10. Nud1 ubiquitination is induced by *DMA2* overexpression preferentially in mitosis and G1

a: Wild type and *GAL1-DMA2* cells expressing Nud1-3PK and overexpressing either untagged ubiquitin or His-tagged ubiquitin were grown in -Trp raffinose medium and induced with galactose for 30’ (cyc) or shifted to YEPR to be arrested in G1 by alpha factor (time 0). G1-synchronised cells were induced with 1% galactose for 30’ before release in YEPRG at 25°C. At the indicated times cells were collected for Ni-NTA pulldown assays, as well as for tubulin immunofluorescence (metaph. sp.: metaphase spindles; anaph. sp.: anaphase spindles). The levels of Pgk1 in the inputs were used as loading controls, while Clb2 was used as a marker of mitosis. We note that under these conditions of ubiquitin overexpression *DMA2* overexpression seems to destabilize Nud1 and slow down mitotic progression. This aberrant Nud1 instability was not observed when *DMA2* was overexpressed in the presence of physiological levels of ubiquitin (see Fig. 5f and Supplementary Fig. 11c). We also note that Nud1 ubiquitination is reproducibly more pronounced in synthetic than in rich medium. **b:** The same cells as in (a) were grown in –Trp raffinose medium and induced with galactose for 30’ (cyc) or shifted to YEPR and arrested in S phase by hydroxyurea (HU) or in mitosis with nocodazole (noc) before being induced with galactose for 30’. Ni-NTA pulldown assays were performed as above.

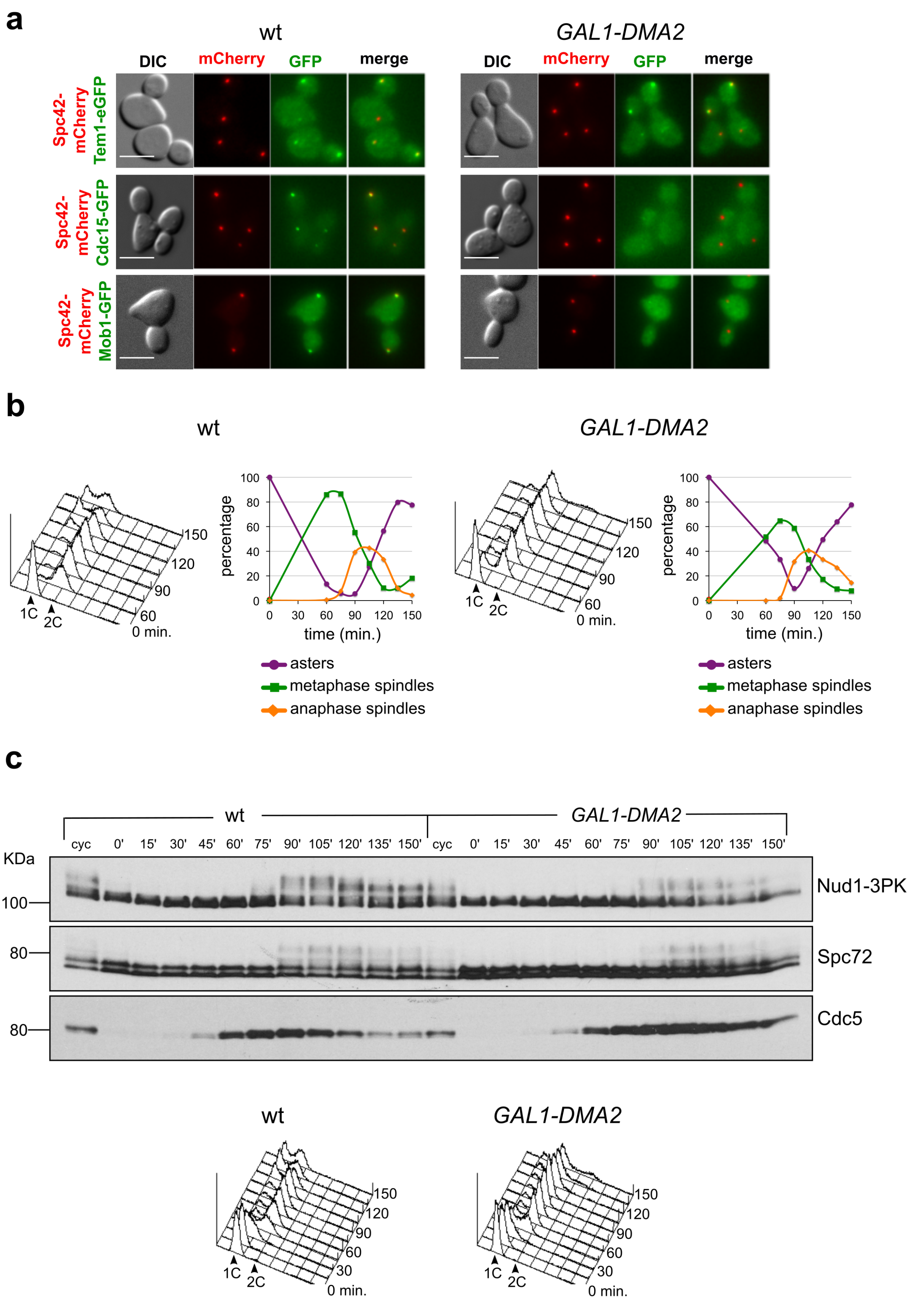

Supplementary Figure 11. Nud1 phosphorylation is impaired upon *DMA2* overexpression
a: Examples of cells expressing Spc42-mCherry and a MEN protein tagged GFP/eGFP from the experiments in Fig. 5d. Scale bar: 5 μm. **b:** FACS analysis of DNA contents (left) and microtubule morphology after *in situ* immunofluorescence of tubulin in wild-type and *GAL1-DMA2* cells relative to the experiment in Fig. 5f. Purple circles: asters; green squares: metaphase spindles; orange diamonds: anaphase spindles. FACS data were plotted after gating out the debris as illustrated in Supplementary Fig. 12.**c:** Wild-type and *GAL1-DMA2* cells expressing Nud1-3PK were grown in YEPR, arrested in G1 with alpha factor and released in fresh YEPRG medium after 30 min induction with galactose. Cells were collected at the indicated times after release (time 0) for FACS analysis of DNA contents (bottom histograms) and for western blot detection of Nud1-3PK, Spc72 and Cdc5. Cyc: cycling cells.

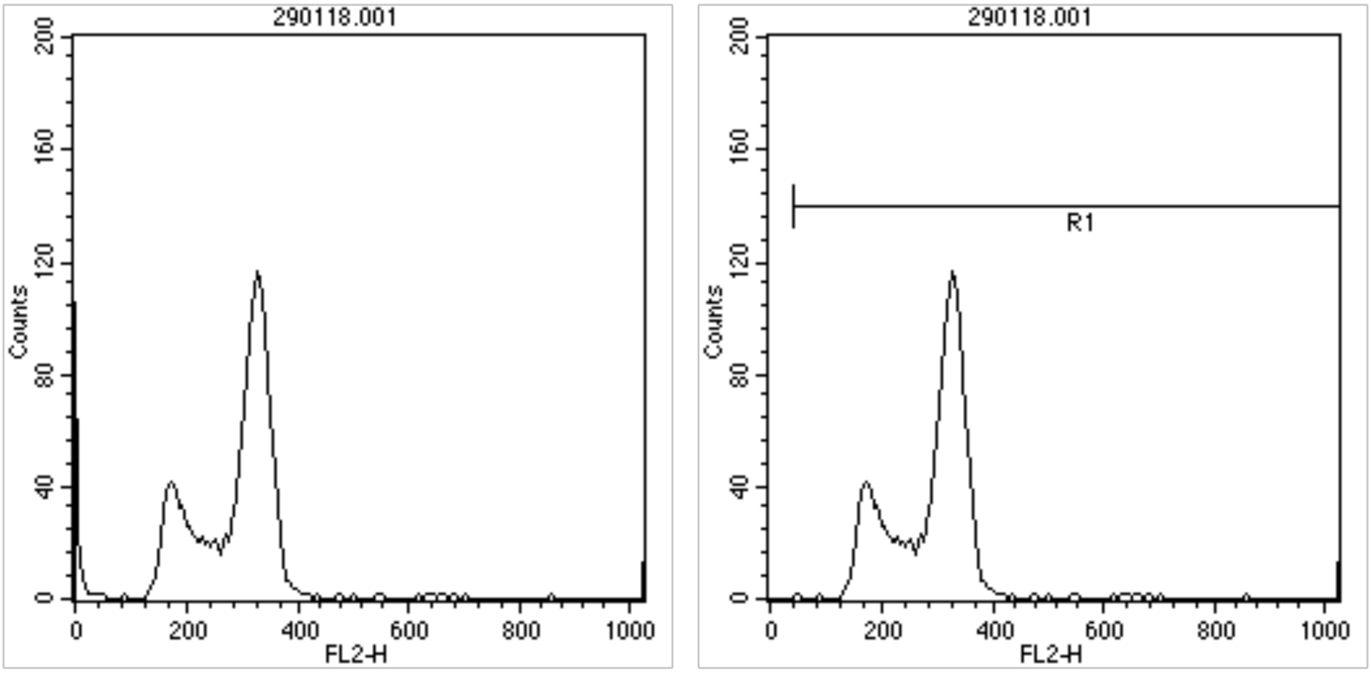

Supplementary Figure 12. Gating strategy applied to all FACS data of this paper
Distribution of DNA contents in an asynchronous population of wild type yeast cells before gating (left histogram) and after gating out the debris with gate R1 (right histogram).

Fig. 5a

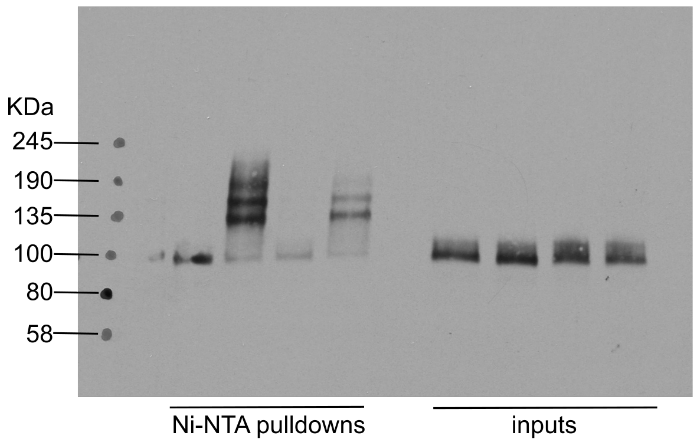

Fig. 5b

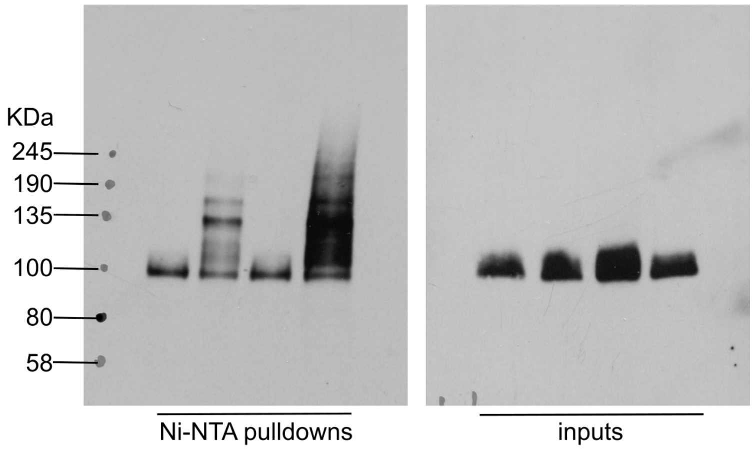

Fig. 5c

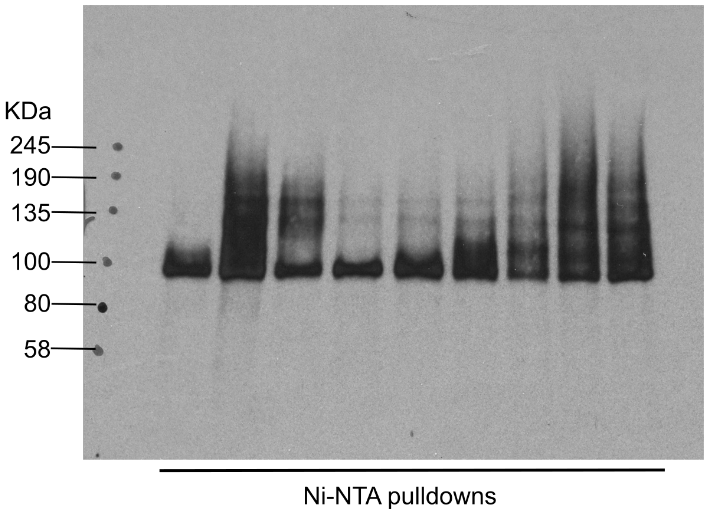

Fig. 5c

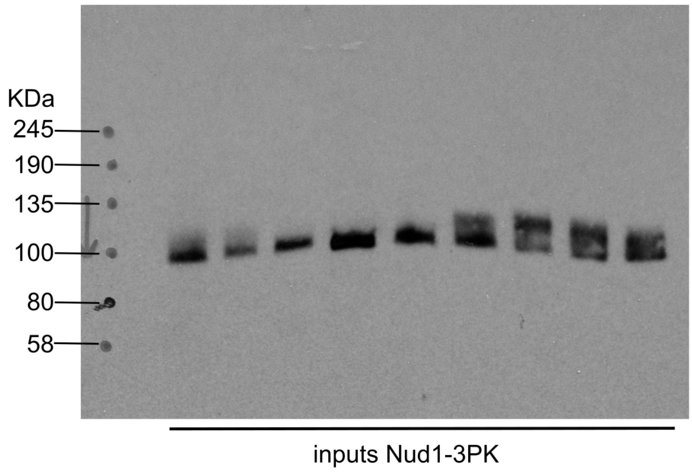

Fig. 5c

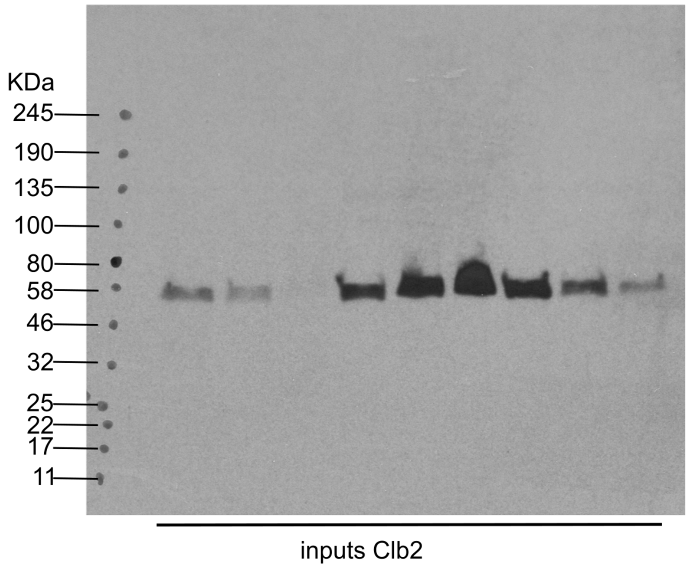

Fig. 5c

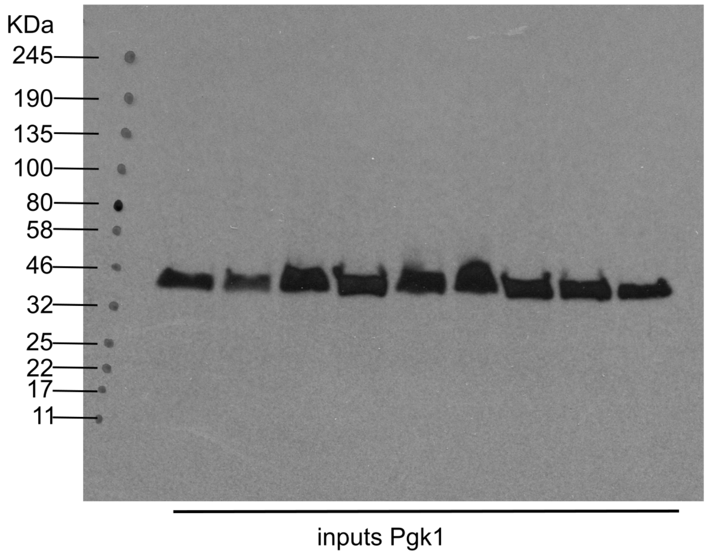

Fig. 5f

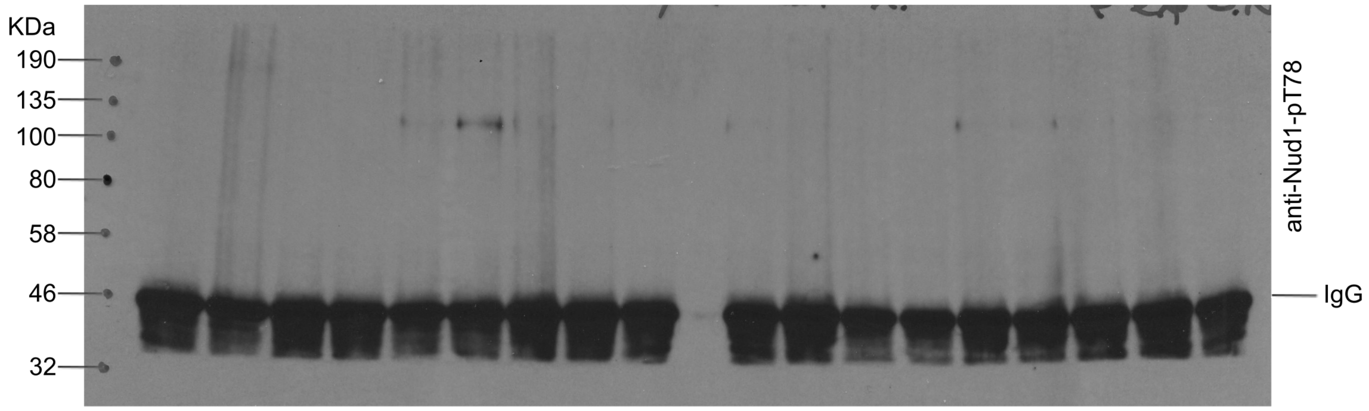

Fig. 5f

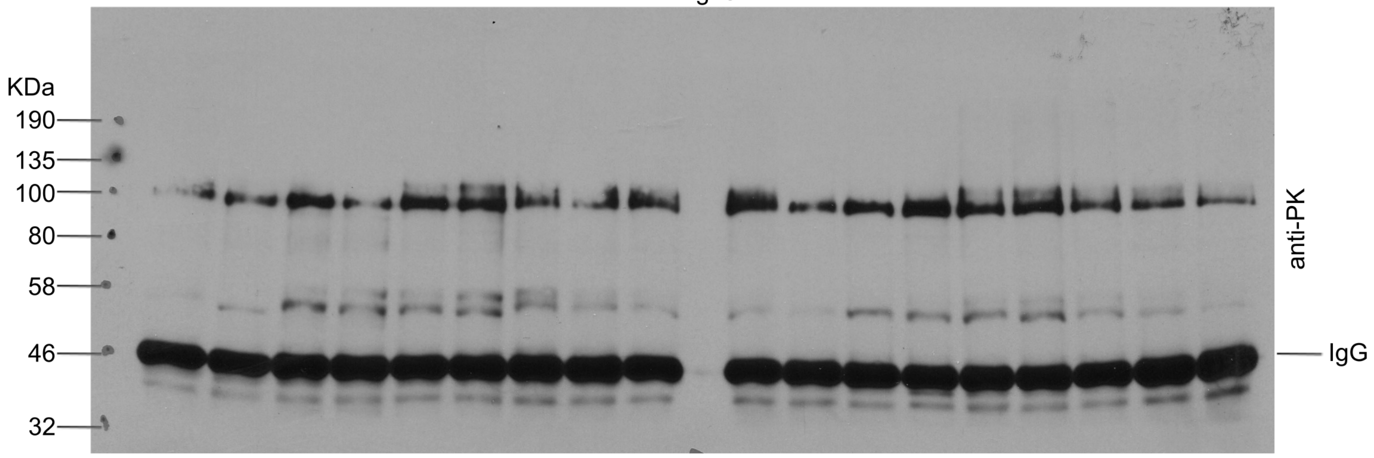

Supplementary Fig. 8a

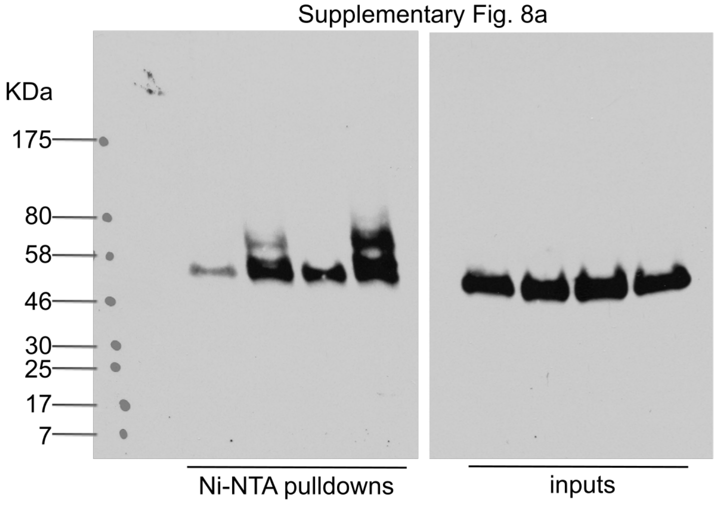

Supplementary Fig. 8b

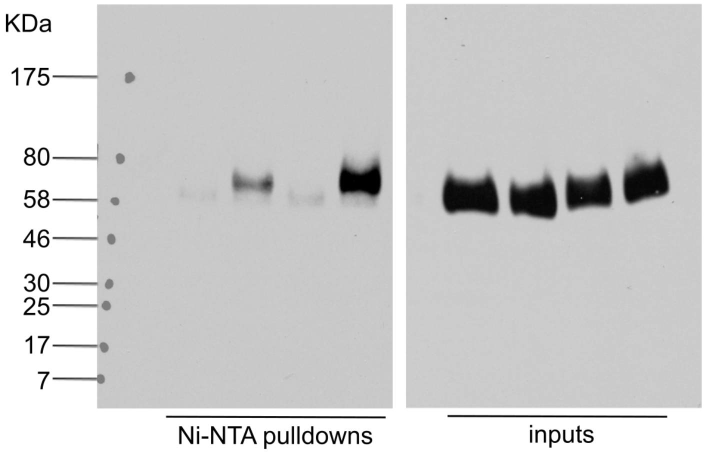

Supplementary Fig. 8c

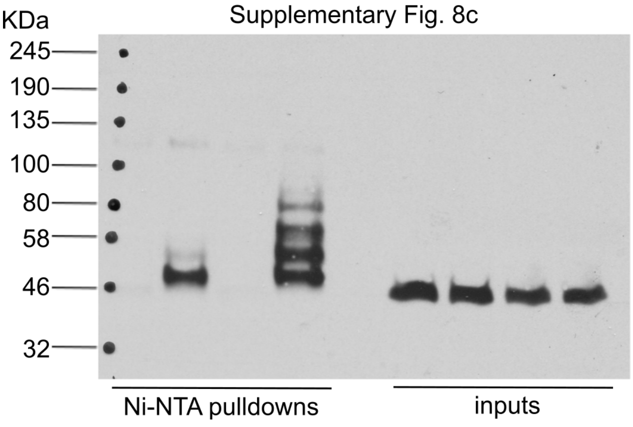

Supplementary Fig. 8d

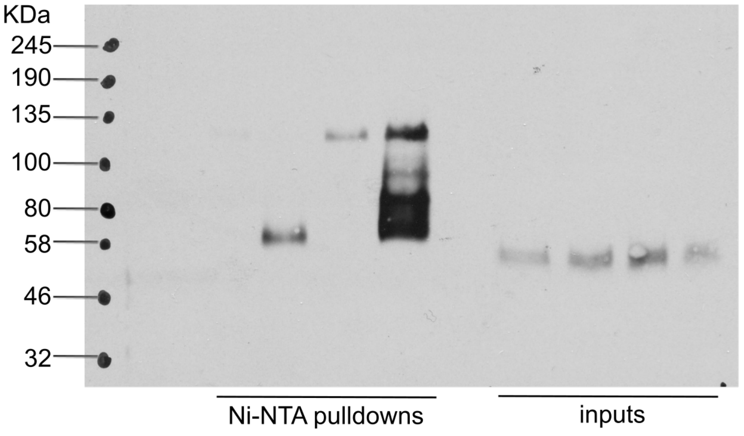

Supplementary Fig. 8e

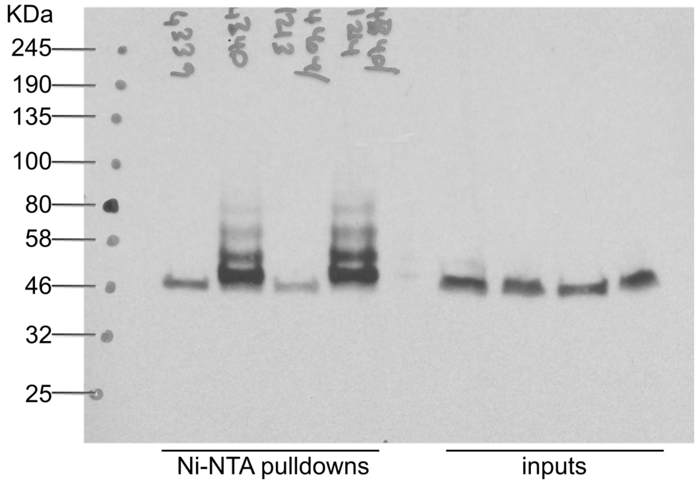

Supplementary Fig. 8f

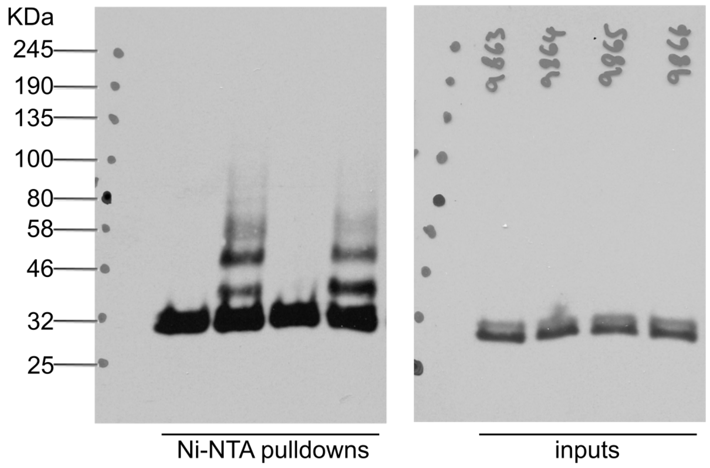

Supplementary Fig. 8g

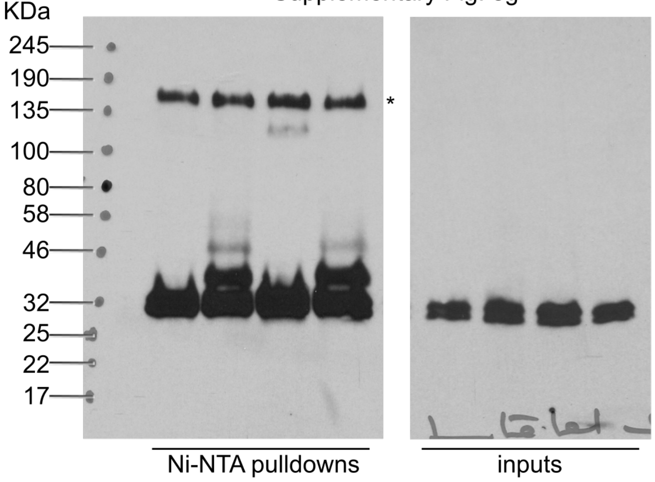

Supplementary Fig. 9

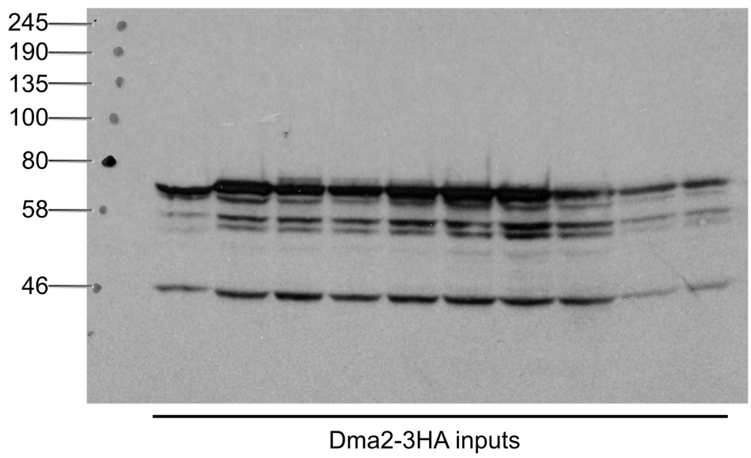

Supplementary Fig. 9

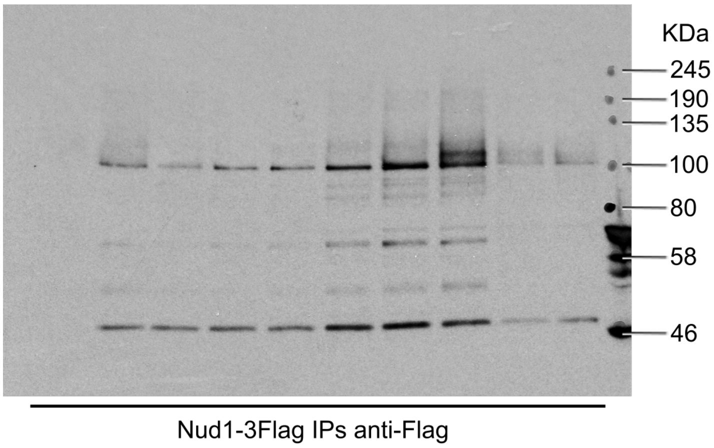

Supplementary Fig. 9

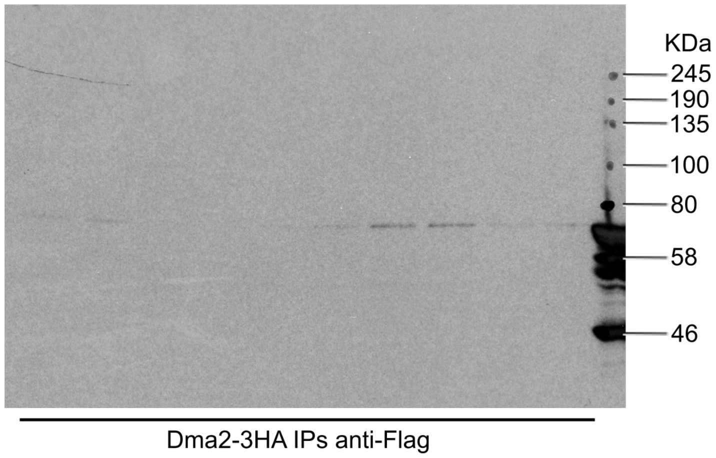

Supplementary Fig. 10a

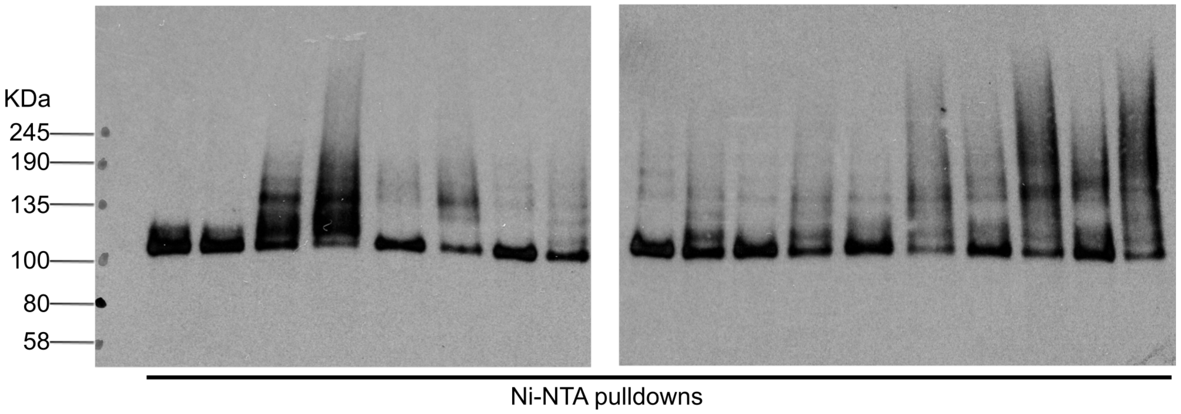

Supplementary Fig. 10a

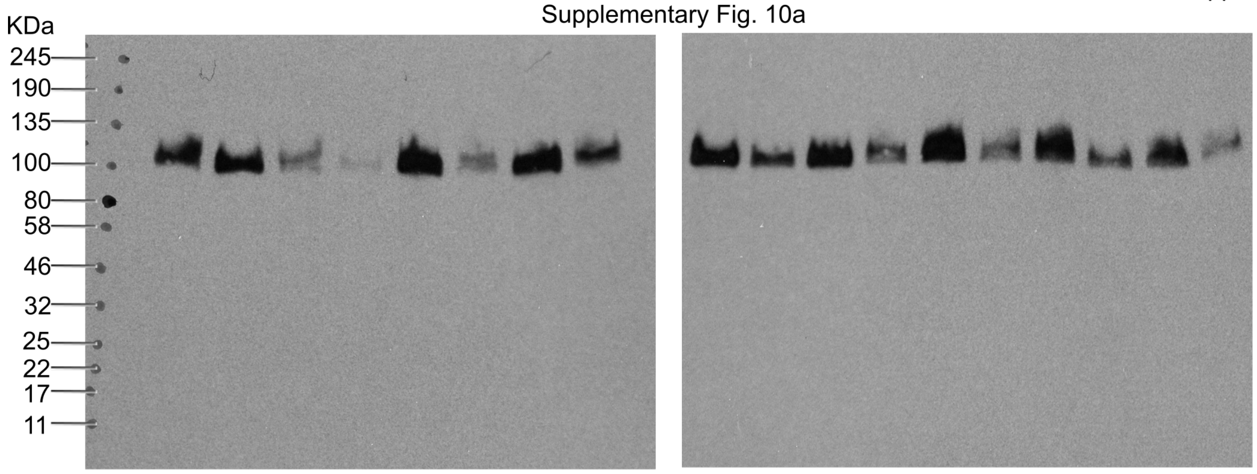

inputs Nud1-3PK

Supplementary Fig. 10a

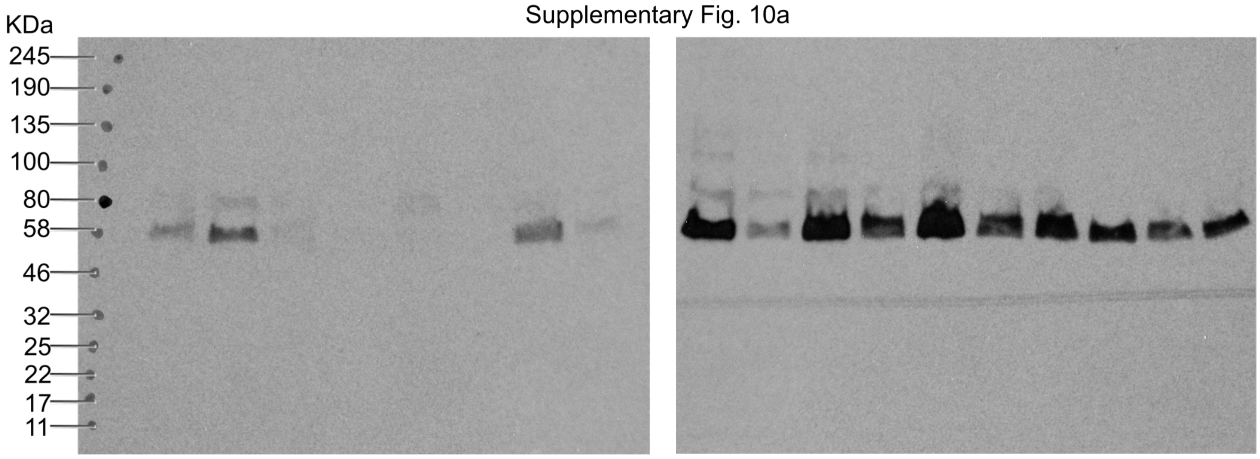

inputs Clb2

Supplementary Fig. 10a

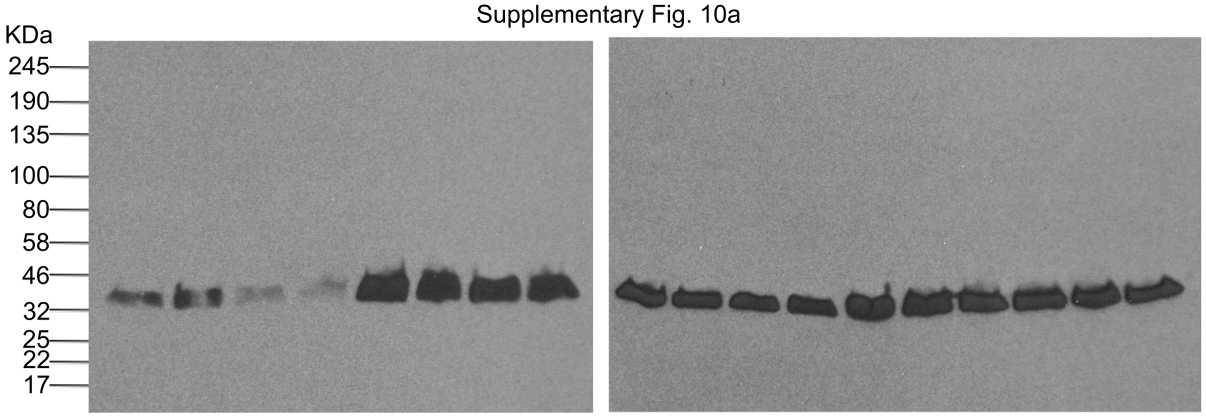

inputs Pgk1

Supplementary Fig. 10b

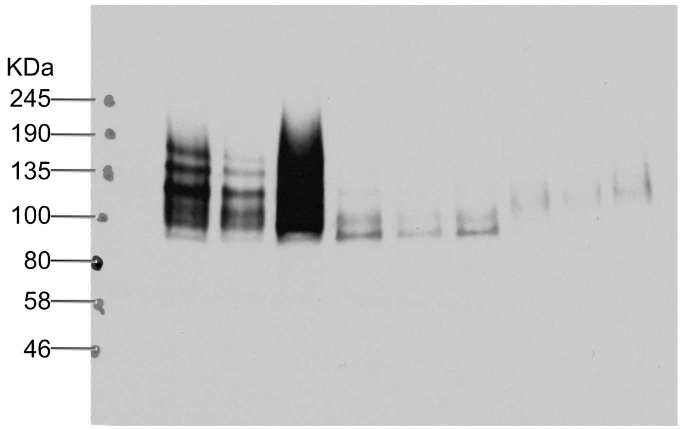

Ni-NTA pulldowns

Supplementary Fig. 10b

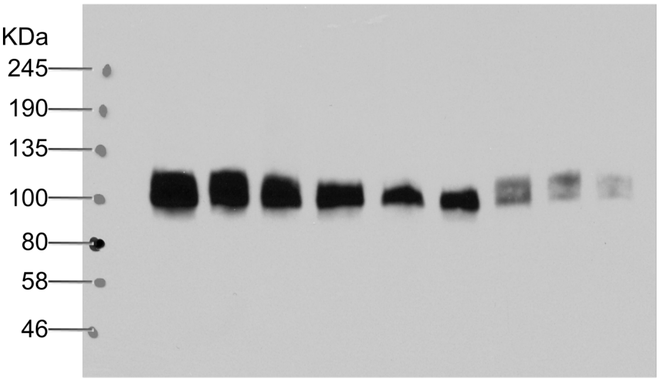

inputs anti-PK

Supplementary Fig. 10b

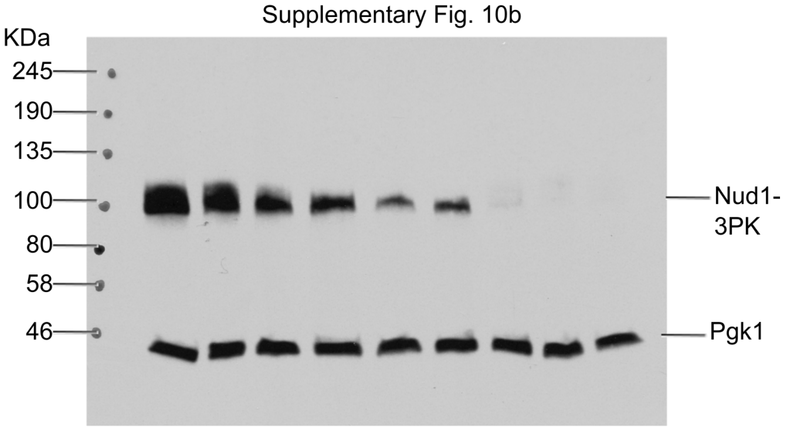

inputs anti-Pgk1

Supplementary Fig. 11c

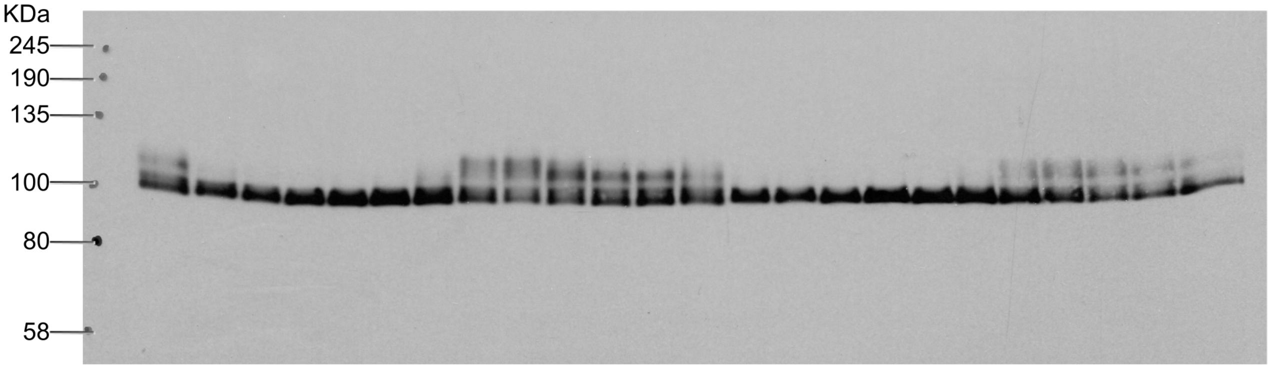

Nud1-3PK

Supplementary Fig. 11c

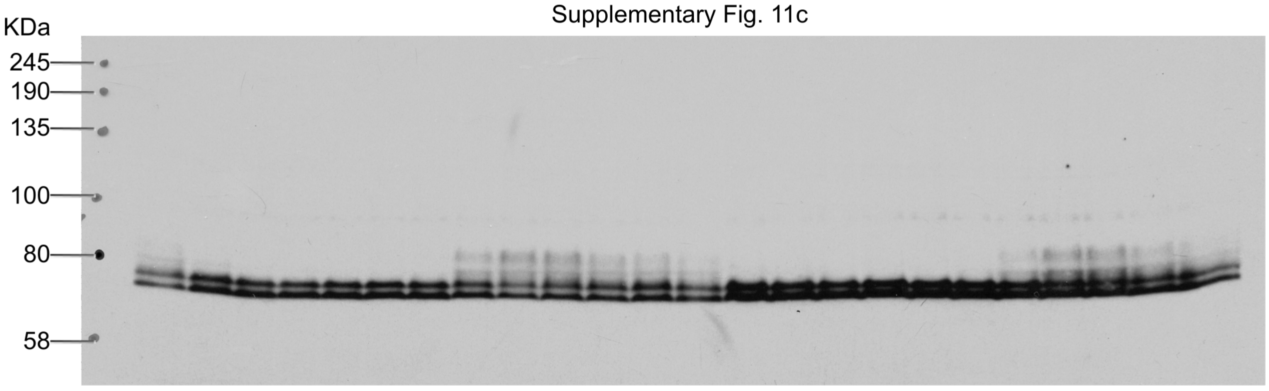

Spc72

Supplementary Fig. 11c

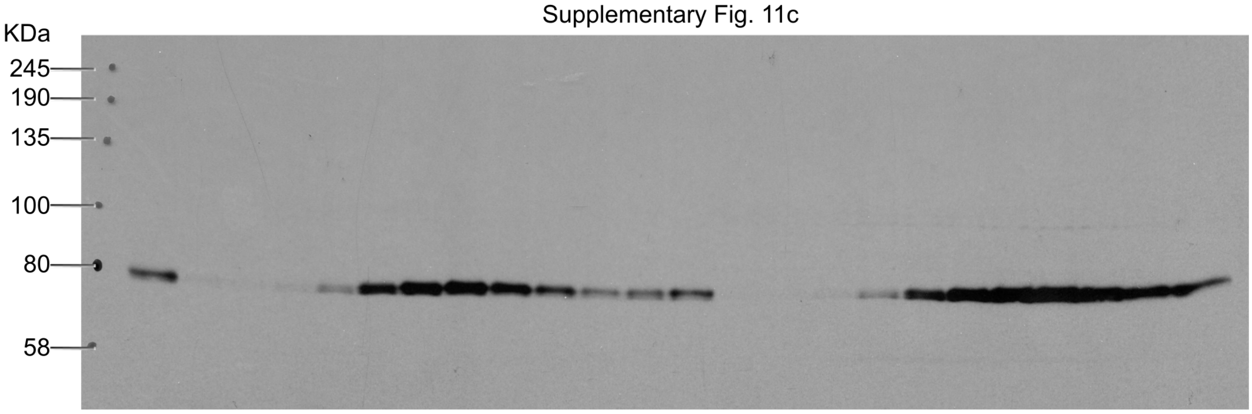

Cdc5

Supplementary References

1. Ko, N. *et al.* Identification of yeast IQGAP (Iqg1p) as an anaphase-promoting-complex substrate and its role in actomyosin-ring-independent cytokinesis. *Mol Biol Cell* **18**, 5139-5153 (2007).
2. Fraschini, R., Bilotta, D., Lucchini, G. & Piatti, S. Functional characterization of Dma1 and Dma2, the budding yeast homologues of Schizosaccharomyces pombe Dma1 and human Chfr. *Mol Biol Cell* **15**, 3796-3810 (2004).
